# The Association Between Bending Photoplethysmography Waveform Area Index and Congestive Heart Failure

**DOI:** 10.1002/clc.70400

**Published:** 2026-07-09

**Authors:** Xianghui Zeng, Qingfeng Zeng, Chunqing Xiao, Hengqing Zhu, Jianping Luo

**Affiliations:** ^1^ Department of Cardiology Ganzhou Hospital of Traditional Chinese Medicine Ganzhou Jiangxi China; ^2^ Department of Cardiology, Ganzhou Hospital‐Nanfang Hospital Southern Medical University (Ganzhou People's Hospital) Ganzhou Jiangxi China; ^3^ Department of Cardiology, Ganzhou Hospital of Guangdong Provincial People's Hospital Ganzhou Municipal Hospital Ganzhou Jiangxi China

**Keywords:** bending, congestive heart failure, diagnostic marker, photoplethysmography waveform area, sitting

## Abstract

**Aims:**

Congestive heart failure (CHF) is closely associated with a decrease in cardiac output, and photoplethysmography (PPG) parameters can experimentally assess cardiac output. It is unclear whether there is an association between the change of bending photoplethysmography waveform area index (BPWAI) due to postural changes and CHF.

**Methods:**

We enrolled patients with suspected CHF. Patients completed physical examination, echocardiography, and pro‐brain natriuretic peptide (NT‐proBNP) test during their visit, and finger‐end PPG waveform area was measured in the sitting and bending positions using PPG. We then used logistic regression to analyze the association between BPWAI and CHF, and the receiver operating characteristic (ROC) curve and area under the curve (AUC) were used to assess the event risk association strength.

**Results:**

A total of 523 patients with suspected CHF were included in this study, of whom 291 (55.6%) had a final diagnosis of CHF. The association between BPWAI and CHF decreased 0.45‐fold for per 1 standard deviation increase in BPWAI (OR: 0.55 [95% CI, 0.44−0.68]). The AUC for BPWAI associated with CHF was 0.736 (95% CI, 0.689−0.782), with a corresponding sensitivity of 67.4% and specificity of 71.5%, and a cut‐off value of 0.85 for the diagnosis of CHF.

**Conclusion:**

When patients bend over, their PPG waveform area decreases, which is closely associated with patients with CHF. BPWAI is significantly associated with CHF and may serve as a potential associated indicator for CHF.

## Graphical abstract

Rather than relying on resting signals alone, BPWAI measures the photoplethysmographic response to bending. This novel dynamic vascular marker was significantly associated with congestive heart failure and may facilitate simple, noninvasive CHF identification in clinical practice.

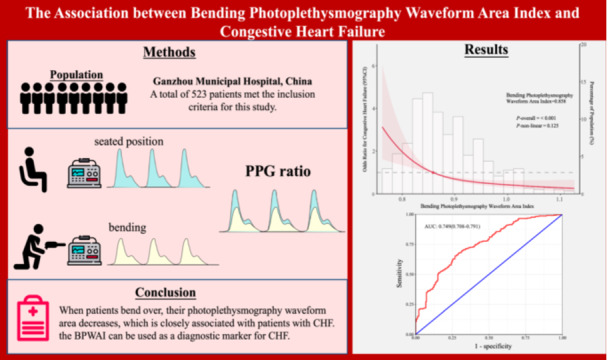

## 1 Introduction

Congestive heart failure (CHF) is the end stage of all types of heart disease and has become an important medical problem due to its high morbidity, mortality, and expense [1]. Rapid identification of CHF is particularly important for early intervention to mitigate disease progression [2]. Currently, the common diagnostic methods for CHF in clinical practice include physical examination, chest X‐ray, echocardiography, and pro‐brain natriuretic peptide (NT‐proBNP) [3]. However, rapid diagnosis of CHF remains a challenge. Finding inexpensive, effective, and convenient screening techniques is of great importance to optimize the diagnosis of CHF.

Several studies have shown that patients with heart failure may have changes in cardiac output due to changes in body position [4, 5, 6]. In 2014, Thibodeau et al. proposed bendopnea, shortness of breath during bending, as a new symptom of advanced heart failure [7]. The position of the heart in the bent position is lower than in the seated position, which increases the return blood volume and also increases the left ventricular preload [7]. However, cardiac autoregulation is impaired in patients with heart failure, and according to the Frank−Starling mechanism, the ejection fraction of the heart decreases instead when preload is increased excessively [8, 9]. Therefore, we ask whether this decrease in cardiac output due to bending postural changes can be used in the diagnosis of CHF. Photoplethysmography (PPG) is a noninvasive test for detecting blood volume changes in living tissue by means of photoelectric [10]. PPG is now widely used in clinical practice because of the rich physiological information [11, 12]. Several studies have reported that PPG can accurately estimate cardiac output [11, 13, 14]. And the PPG area is closely related to cardiac output [15]. Based on the above principles, this study was used to identify CHF by examining the PPG area of subjects in bending and sitting separately and then calculating the ratio of the two. This study would provide an economical, simple, and rapid noninvasive method for the diagnosis of CHF.

## 2 Methods

### 2.1 Study Population

A single‐center cross‐sectional study was conducted in patients with suspected CHF (the main symptoms were dyspnea and lower limb edema) who presented to Ganzhou Municipal Hospital, China, between April 2018 and August 2020. The study was approved by the Research Ethics Committee of Ganzhou Municipal Hospital, China (Ethics number: 2018006), and was based on the principles outlined in the Declaration of Helsinki. The study procedures were fully explained to each patient, and consent was obtained and signed by the investigators before the tests were performed.

Subjects were eligible if they were older than 20 years of age and able to provide written informed consent. Exclusion criteria: (1) no documented echocardiogram and NT‐proBNP test during the consultation; (2) acute left heart failure (defined as evidence of acute pulmonary edema, hypoxemia on the day of enrollment); (3) severe pulmonary disease requiring home oxygenation; (4) previous mechanical circulatory support with a heart transplant or ventricular assist device; (5) uncontrolled arrhythmias (sustained ventricular tachycardia or atrial fibrillation/flutter); (6) patients who are unable to bend forward for non‐HF reasons such as musculoskeletal problems. CHF was diagnosed based on (1) typical symptoms (dyspnea, edema), (2) clinical signs, (3) echocardiographic evidence of structural or dysfunction, and (4) elevated NT‐proBNP, according to guidelines for the diagnosis and treatment of acute and chronic heart failure [16]. CHF was diagnosed by two independent physicians [17].

### 2.2 PPG Waveform Area Definition

**Figure 1:**
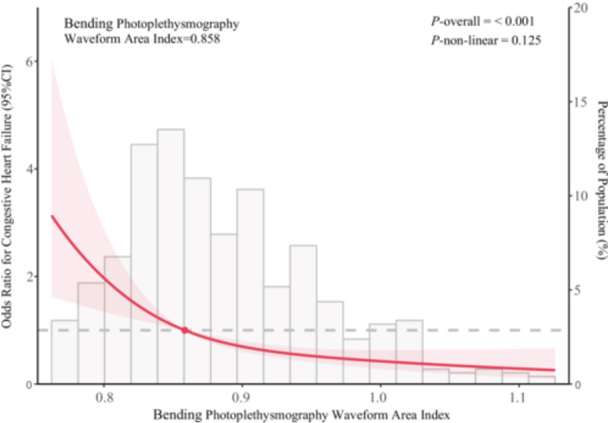
Adjusted spline curves for the association of bending photoplethysmography waveform area index with congestive heart failure. The model was adjusted for age, sex, BMI, heart rate, smoke, stroke, coronary atherosclerotic heart disease, chronic obstructive pulmonary disease, diabetes, hypertension, chronic kidney disease, antihypertension, hypoglycemic. The solid and dashed lines indicate the estimated OR and 95% CI, respectively. BMI, body mass index; CI, confidence interval; OR, odds ratios.

**Figure 2:**
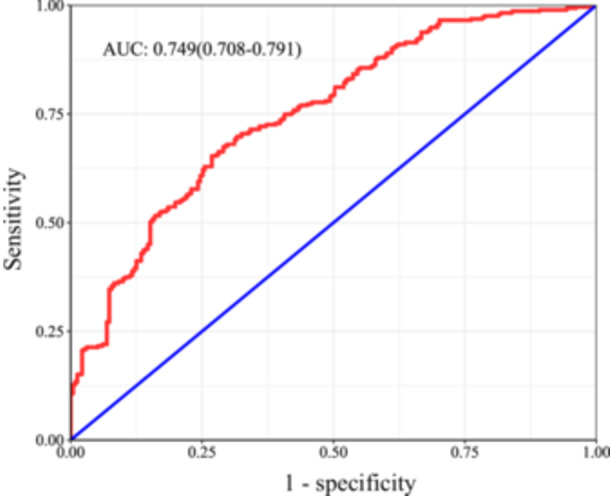
The receiver operating characteristic (ROC) curves of the bending photoplethysmography waveform area index for the association with congestive heart failure. AUC, area under the curve; ROC, receiver operating characteristic.

Before testing the PPG waveform area, it was confirmed that all subjects could tolerate the operation in the bent position for 30 s. To avoid measurement errors, all measurements were made to ensure that the finger end was warm, clean and well perfused, and patients were asked to place the hand with the finger probe on a stable surface to minimize motion interference and without interference from other equipment (Supporting Information S1: Figure 1). The subject was kept in a seated position, and five consecutive PPG waveforms were recorded after the graph was stabilized, after which the subject was instructed to bend forward at the waist, as if tying a shoelace [18]. During bending, five consecutive PPG waveforms were recorded again after the graph was stabilized. All PPG recordings were performed using the Goldway UT4000F (Shenzhen, China). The operation was performed by the same two trained physicians who were blinded to the clinical diagnosis and clinical data (Supporting Information S1: Figure 2).

PPG recordings were visually inspected for signal quality, and recordings with excessive motion artifacts were excluded. Five consecutive stable pulse waveforms were selected from each recording. The waveform area of each pulse was calculated as the area under the PPG waveform between two consecutive pulse wave onsets. The mean waveform area of the five selected pulses was used as the representative waveform area for the corresponding posture. The bending photoplethysmography waveform area index (BPWAI) was calculated as the ratio of the mean waveform area in the bending position to that in the sitting position.

### 2.3 Variates

A questionnaire was used to collect demographic information (age, gender), smoking (smoking status according to a self‐reported questionnaire [current smokers were defined as those who had smoked ≥ 100 cigarettes in their lifetime and within the past 3 months, former smokers were those who had smoked ≥ 100 cigarettes in their lifetime and last smoked more than 3 months ago]), medical history (diabetes, coronary heart disease, stroke, hypertension, chronic obstructive lung disease [history of previous diagnosis by a physician]) and history of current medications (antihypertensive medications and blood glucose lowering medications). Body mass index (BMI) was calculated according to the formula BMI = weight (kg)/height (m) ^2^.

In addition, NT‐proBNP and echocardiographic data (left ventricular ejection fraction [LVEF]) obtained during the visit were collected. Echocardiography was performed by an experienced sonographer using a comprehensive transthoracic echocardiography using a vivid color echodoppler diagnostic instrument manufactured and provided by GE.

### 2.4 Statistical Analysis

The BPWAI was grouped into tertiles. Categorical variables were expressed as cases and percentages; continuous variables were represented as median and interquartile range (IQR), and categorical variables were analyzed by chi‐square test for differences between groups, and continuous variables were compared between groups by rank sum test.

Logistic regression models were used to estimate the risk ratio (OR) and 95% confidence interval (CI) of the association between BPWAI and CHF. Covariates were selected based on clinical relevance, established pathophysiological evidence, and prior peer‐reviewed literature related to CHF and PPG waveform characteristics [19, 20, 21]. To systematically account for potential confounders, three covariate models were constructed: model 1 was unadjusted; model 2 was adjusted for age and sex; model 3 was further adjusted for model 2 plus BMI, heart rate, smoke, stroke, coronary atherosclerotic disease (CAD), chronic obstructive pulmonary disease (COPD), diabetes mellitus, hypertension, chronic kidney disease (CKD), and antihypertension, hypoglycemic. The linear trend was tested by a median value for each tertile as a continuous variable. In addition, the continuous exposure variable (BPWAI) was modeled using restricted cubic spline regression to examine the potential linear relationship between BPWAI and CHF. Participant operating characteristic (ROC) curves and area under the curve (AUC) adjusted for all covariates were used to assess the association strength between BPWAI and CHF. All analyses were performed using R software (version 4.2.0). Two‐sided *p* < 0.05 was considered statistically significant.

## 3 Results

A total of 523 patients met the inclusion criteria for this study. The mean age of the patients was 69 years, 59.5% were male, and 291 (55.6%) had a diagnosis of CHF. The mean BPWAI was 0.87. Patients were divided into three groups according to BPWAI levels: low, medium, and high (BPWAI < 0.85, 0.85 ≤ BPWAI < 0.908, and BPWAI ≥ 0.908). Significant between‐group differences were observed in BMI, hypertension, CAD, use of antihypertensive drugs, heart rate, NT‐proBNP, and LVEF (all *p* < 0.05), indicating baseline imbalance across BPWAI categories. Patients with CHF were more likely to have hypertension, CKD, CAD, LVEF ≤ 50%, and high NT‐proBNP (Table 1).

**Table 1:** Baseline characteristics of participants according to bending photoplethysmography waveform area index.

		BPWAI levels						
Characteristic	Total	Low (< 0.849)	Middle (0.849−0.908)	High (≥ 0.908)	*p* value	Non‐CHF	CHF	*p* value
	*N* = 523	*N* = 174	*N* = 174	*N* = 175		*N* = 232	*N* = 291	
Age, years	69 (60, 74)	67 (61, 73)	70 (62, 74)	68 (55, 74)	0.18	66.0 (57.0, 73.0)	71.0 (62.0, 74.0)	< 0.01
Sex					0.15			0.18
Male	311 (59.5)	113 (64.94)	102 (58.62)	96 (54.86)		146 (62.93)	165 (56.7)	
Female	212 (40.5)	61 (356)	72 (41.38)	79 (45.14)		86 (37.07)	126 (43.3)	
BMI, kg/m^2^	25.9 (22.2, 29.7)	23.3 (21.2, 24.6)	28.4 (27.1, 29.7)	23.3 (21.3, 34)	< 0.01	27.5 (23.6, 31.1)	24.5 (21.9, 8.05)	< 0.01
Heart rate, bpm	84 (70, 99)	87 (74, 98)	80 (71, 93)	71 (64, 78)	0.025	73 (63, 84)	90 (77, 105)	< 0.01
Smoking status					0.24			< 0.01
Never	270 (51.6)	96 (55.17)	90 (51.72)	84 (48)		94 (40.52)	176 (60.48)	
Former	180 (34.4)	49 (28.16)	63 (36.21)	68 (38.86)		90 (38.79)	90 (30.93)	
Now	73 (14)	29 (16.67)	21 (127)	23 (13.14)		48 (20.69)	25 (8.59)	
Baseline comorbidities								
Stroke	38 (7.3)	9 (5.17)	16 (9.20)	13 (7.43)	0.35	24 (10.34)	14 (4.81)	0.02
CAD	176 (14.5)	103 (58.86)	55 (31.6)	38 (21.83)	0.03	39 (16.81)	157 (53.95)	< 0.01
CKD	93 (17.8)	29 (16.67)	33 (18.97)	31 (17.71)	0.85	28 (12.07)	65 (22.34)	< 0.01
COPD	32 (6.1)	8 (4.60)	9 (5.17)	15 (8.57)	0.25	26 (11.21)	6 (2.06)	< 0.01
Diabetes	173 (33.1)	43 (24.71)	64 (36.78)	66 (37.71)	0.16	86 (37.07)	87 (29.9)	0.10
Hypertension	357 (68.3)	107 (61.49)	136 (78.16)	114 (65.14)	0.02	109 (46.98)	248 (85.22)	< 0.01
Medications used								
Antihypertension^a^	281 (53.7)	75 (43.10)	115 (66.9)	91 (52)	< 0.01	75 (32.33)	206 (70.79)	< 0.01
Hypoglycemic^b^	138 (26.4)	36 (20.69)	50 (28.74)	52 (29.71)	0.1108	69 (29.74)	69 (23.71)	0.15
NT‐proBNP, pg/mL	880.0 (150.0, 1600.0)	1520.0 (290.0, 2100.000)	800.0 (220.0, 1450.0)	195.0 (95.0, 950.0)	< 0.01	140.0 (95.0, 220.0)	1520.0 (1050.0, 2100.0)	< 0.01
LVEF					< 0.01			< 0.01
LVEF ≤ 40%	145 (27.72)	64 (50.8)	44 (20.75)	38 (20.3)		0 (0.0)	145 (49.82)	
40% < LVEF ≤ 50%	61 (11.66)	31 (25.0)	16 (7.54)	14 (7.5)		7 (3.02)	54 (18.57)	
LVEF > 50%	317 (60.61)	30 (24.19)	152 (71.7)	134 (72.22)		225 (96.98)	92 (31.62)	

*Note:* Values are median and interquartile range.

Abbreviations: BMI, body mass index; BPWAI, photoplethysmography waveform area index; CAD, coronary atherosclerotic disease; CHF, congestive heart failure; CKD, chronic kidney disease; COPD, chronic obstructive pulmonary disease; LVEF, left ventricular ejection fraction; NT‐proBNP, pro‐brain natriuretic peptide.

^a^Antihypertension included angiotensin‐converting enzyme inhibitors, angiotensin receptor blockers, calcium antagonists, beta‐blockers, and diuretics.

^b^Hypoglycemic included oral hypoglycemic drugs and insulin.

In the model without adjusting for covariates, compared to patients with the low BPWAI levels, the ORs of CHF were observed to be 0.57 (95% CI, 0.37−0.89) and 0.27 (95% CI, 0.17−0.42) for patients with the middle BPWAI levels or the high BPWAI levels, respectively (Table 2). The full‐adjusted ORs (Model 3) of CHF were 0.52 (95% CI, 0.32−0.85) and 0.26 (95% CI, 0.16−0.43) for patients with the middle BPWAI levels or the high BPWAI levels compared to the low BPWAI levels, respectively (Table 2). In addition, for a per‐SD (0.083) increase in BPWAI, the ORs for CHF were 0.55 (95% CI, 0.45−0.67) and 0.55 (95% CI, 0.44−0.68) for patients with the middle BPWAI levels or the high BPWAI levels compared to the low BPWAI levels, respectively (Table 2).

**Table 2:** Odds ratios (95% CIs) for risk of congestive heart failure according to bending photoplethysmography waveform area index.

		OR (95% CI)		
	**Total/event**	**Model 1**	**Model 2**	**Model 3**
BPWAI				
Low	174/54	1 (ref)	1 (ref)	1 (ref)
Middle	174/94	0.57 (0.37, 0.89)	0.57 (0.36, 0.89)	0.46 (0.28, 0.75)
High	175/84	0.27 (0.17, 0.42)	0.28 (0.18, 0.44)	0.25 (0.15, 0.41)
*p* for trend	—	< 0.0001	< 0.0001	< 0.0001
Per 1 SD	—	0.55 (0.45, 0.67)	0.56 (0.46, 0.68)	0.55 (0.44, 0.69)

*Note:* Model 1: no variables are adjusted.

Model 2: age and sex.

Model 3: age, sex, BMI, heart rate, smoke, stroke, coronary atherosclerotic disease, chronic obstructive pulmonary disease, diabetes, hypertension, chronic kidney disease, antihypertension, and hypoglycemic.

Abbreviations: BMI, body mass index; BPWAI, bending photoplethysmography waveform area index; CI, confidence interval; OR, odds ratios; Ref, reference; SD, standard deviation.

Stratified analysis of age (< 65, ≤ 65 years), sex (male, female), or BMI (< 25 kg/m^2^, ≤ 25 kg/m^2^) with data given in Supporting Information S1: Table 1. There was no evidence that the association between BPWAI levels and all‐cause and CHF differed by BMI, age, or sex.

The dose−response relationship analysis of BPWAI and CHF was shown in Figure 1. The BPWAI showed a linear relationship with CHF (*P* for non‐linearity = 0.125). The prevalence of CHF increased significantly with decreasing BPWAI. AUC for BPWAI for the association with CHF was 0.7495 (95% CI: 0.7081−0.7909), with a corresponding sensitivity of 67.4% and specificity of 71.5%, and a cut‐off value of 0.85 for the diagnosis of CHF (Figure 2).

## 4 Discussion

In this cross‐sectional study of patients with suspected heart failure, we found that a decreased bending PPG waveform area ratio was strongly associated with CHF. To our knowledge, this is the first study to explore the relationship between BPWAI and CHF during postural changes. Our study showed that lower BPWAI was strongly associated with CHF, and a threshold of 0.85 showed good discriminatory ability. The results of this study provide a potentially useful associated indicator for identifying patients at risk of CHF, and the method is simple, economical, and quick.

This pulsating change in blood volume in microvessels in response to heartbeats is called a volumetric pulse wave [11]. PPG is one of the most commonly used methods for extracting pulse wave signals, as it provides an accurate picture of blood flow changes in microvessels. PPG is commonly used to determine arterial oxygen saturation, pulse rate, and respiratory rate, among others [22]. Pulse monitoring by a wearable device can screen for atrial fibrillation [23]. The previous study by our team showed that PPG measurement of pulse wave conduction velocity can diagnose atherosclerosis [24]. Sanyal et al. showed that photoelectric volumetric pulse wave imaging can also be used for contactless assessment of respiratory rate [25]. PPG can also estimate arterial oxygen saturation by calculating oxyhemoglobin and deoxyhemoglobin levels [26]. In summary, PPG has been widely used in the medical field, and its technology is mature and easy to operate.

A reduced bending PPG waveform area was strongly associated with CHF in our study. Bendopnea, defined as shortness of breath within 30 s after bending forward while sitting, is the latest symptom in the classic HF symptom portfolio [7]. It is thought to be mediated by an increase in ventricular filling pressures during bending, which exacerbates the already high filling pressures in patients with HF [27]. The position of the heart in the bending position is lower than in the sitting position, which increases the amount of return blood and also increases the LV filling preload [7]. In the bent‐over position, the abdominal cavity is compressed, and the diaphragm is elevated resulting in increased thoracic pressure. The heart is compressed, thus increasing the left ventricular preload [7, 28, 29]. However, cardiac autoregulation is impaired in patients with heart failure, and according to the Frank−Starling mechanism, LVEF decreases instead when the current load is increased excessively [8, 9]. In this study, we cleverly used PPG to detect changes in cardiac output during bending in heart failure, identifying patients with and without heart failure. PPG can accurately assess changes in peripheral microcirculatory blood flow [30]. Alian et al. found that PPG changes can be used as an early indicator of blood loss [15]. The study by Wijshoff et al. found that cardiac output changes could be assessed by calculating the PPG waveform area to monitor cardiac output in patients undergoing cardiopulmonary resuscitation [31].

PPG is widely available, inexpensive, noninvasive, and has been used as a routine patient monitoring technology for many years, easily accessible from existing patient monitoring devices or inexpensive instruments. It is also simple to operate and suitable for use by non‐medical technicians for home healthcare, and can also be extended with networking capabilities and telemedicine, allowing the formation of medical networks for home, double stations, and hospitals, and the implementation of remote medical monitoring, which has good prospects for application [12, 32]. Our findings suggest that a decrease in PPG waveform area during bending may serve as a simple, noninvasive indicator associated with CHF for preliminary clinical evaluation.

Several limitations need to be acknowledged. First, this study has the inherent limitations of being a single‐center study, and the sample size of this study was relatively small. Although statistically significant changes in PPG were confirmed, validation in a multicenter, larger study population is still needed. Second, this study used a cross‐sectional design, which only demonstrates an association rather than diagnostic or predictive causality. Therefore, the results should be interpreted as an association rather than definitive diagnostic evidence. Third, baseline imbalance was noted in BMI, hypertension, CAD, heart rate, NT‐proBNP, and LVEF across groups, reflecting real‐world clinical characteristics. Although a comprehensive multivariable adjustment was applied, residual confounding could not be fully excluded. Nevertheless, the association between lower BPWAI and CHF remained robust and statistically significant in all adjusted models. Fourth, finger PPG signals are susceptible to contact forces and local vascular tone, and more extensive testing is needed to explore the impact of these issues and optimize their performance [33, 34]. Finally, we did not perform invasive hemodynamic assessments, so we were unable to establish a direct correlation between BPWAI and invasive cardiac output.

## 5 Conclusion

This study shows that decreased BPWAI in the bent position is closely related to CHF and that bent PPG area/sitting PPG area may serve as a potential indicator associated with CHF. Given that PPG is inexpensive, noninvasive, and widely available in wearable devices, BPWAI may have potential value for home‐based screening and remote monitoring of patients at risk of heart failure. However, because this study did not assess quality of life or long‐term outcomes, future prospective studies are needed to determine whether BPWAI‐guided monitoring can improve patient management and quality of life.

## Author Contributions

The authors' responsibilities were as follows: Hengqing Zhu had full access to all of the data in the study and took responsibility for the integrity of the data and the accuracy of the data analysis. Concept and design: Hengqing Zhu. Acquisition, analysis, or interpretation of data: All authors. Drafting of the manuscript: Qingfeng Zeng and Xianghui Zeng. Critical revision of the manuscript for important intellectual content: Jianping Luo and Hengqing Zhu. Statistical analysis: Qingfeng Zeng, Hengqing Zhu, and Xianghui Zeng. Administrative, technical, or material support: Qingfeng Zeng, Jianping Luo, and Xianghui Zeng. Supervision: Jianping Luo. All authors approved the final version of the manuscript.

## Ethics Statement

The study was approved by the Research Ethics Committee of Ganzhou Municipal Hospital, China, and was based on the principles outlined in the Declaration of Helsinki. The study procedures were fully explained to each patient, and consent was obtained and signed by the investigators before the tests were performed.

## Consent

The study procedures were fully explained to each patient, and consent was obtained and signed by the investigators before the tests were performed. Participants/patients gave written informed consent for their personal or clinical details, along with any identifying images, to be published in this study.

## Conflicts of Interest

The authors declare no conflicts of interest.

## Supporting information

Supporting File

## Data Availability

The data that support the findings of this study are available on request from the corresponding author. The data are not publicly available due to privacy or ethical restrictions.
