# Supplementary material for: The Association Between Bending Photoplethysmography Waveform Area Index and Congestive Heart Failure
Source: Clin Cardiol. 2026 Jul 9;49(7):e70400. doi: 10.1002/clc.70400 (PMC13348781; doi:10.1002/clc.70400)
Supplement: Supplementary file 1 — Supporting File [file CLC-49-e70400-s001.pdf]

Supplementary Online Content

Supplement Figure 1. Schematic diagram of PPG measurement at finger end.

Supplement Figure 2. Correlation analysis between the first BPWAI (1nd BPWI) and the second BPWAI (2nd BPWAI).

Supplement Table 1. Stratified Analyses of the Associations between Bending Photoplethysmography Waveform Area Index and Congestive Heart Failure.

This supplementary material has been provided by the authors to give readers additional information about their work.

Supplement Figure 1. Schematic diagram of PPG measurement at finger end.

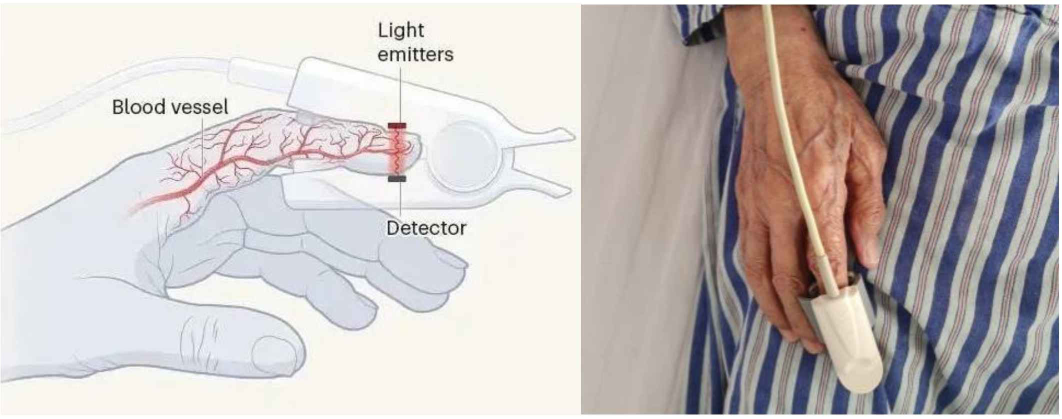

We used a finger-end sensor clip to capture PPG waveforms. Pressure on each participant's skin was relatively constant with the fingertip sensor. The fingertip was placed flat at the thigh in the sitting position and bent over with the finger at the ankle.

Supplement Figure 2. Correlation analysis between the first BPWAI (1nd BPWI) and the second BPWAI (2nd BPWAI).

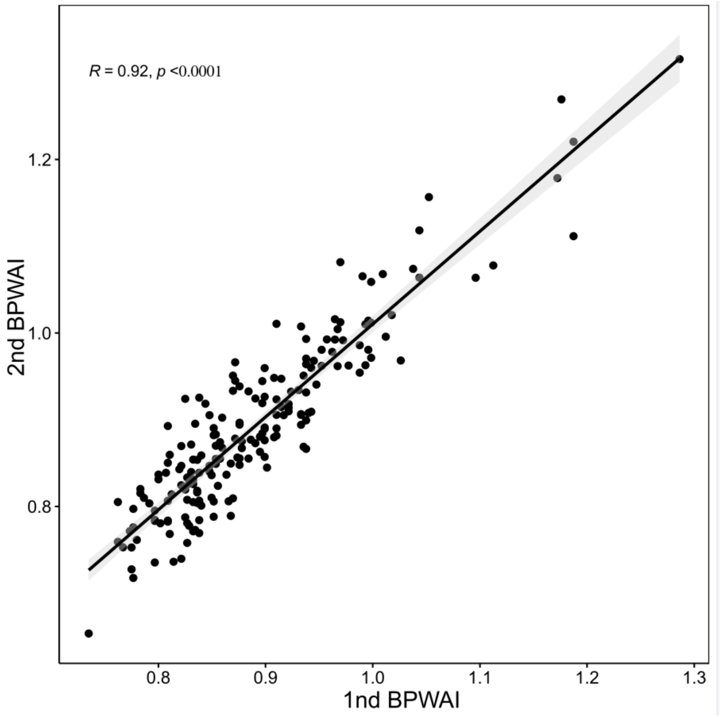

Participants were seated or in a bent-over position, and after the waveform had stabilized, participants were instructed to hold their breath after a deep inhalation. We recorded PPG waveforms greater than 15 seconds and then selected 5 consecutive PPG waveforms to measure (Calculate the average area of the 5 PPG waveforms). Only 36.52% of participants did a second measurement of the PPG waveform 10 minutes after completing the first measurement. We performed a correlation analysis of these two BPWAIs and visualized them using scatter plots. The results suggested that the two BPWAIs correlated well (R = 0.92, p < 0.0001).

Supplement Table 1. Stratified Analyses of the Associations between Bending Photoplethysmography Waveform Area Index and Congestive Heart Failure.

	**BPWAI levels**			
**Characteristic**	**Low**	**Middle**	**High**	***P*-interaction**
**Age, year**				0.9674
<65	1(ref)	0.63(0.24, 1.17)	0.24(0.11, 0.52)	
65≤	1(ref)	0.52(0.26, 0.99)	0.26(0.13, 0.50)	
**Sex**				0.3979
Male	1(ref)	0.41(0.21, 0.77)	0.28(0.15, 0.53)	
Female	1(ref)	0.67(0.29, 1.58)	0.19(0.08, 0.44)	
**BMI, kg/m^2**				0.8968
<25	1(ref)	0.51(0.21, 1.23)	0.2(0.08, 0.46)	
25≤	1(ref)	0.54(0.29, 1)	0.25(0.13, 0.46)	

Data are shown in OR and 95%CI.

The model was adjusted for age, sex, BMI, smoke, stroke, heart rate, coronary atherosclerotic disease, chronic obstructive pulmonary disease, diabetes, hypertension, chronic kidney disease, antihypertension, hypoglycemic, except for the sub-group variable.

Abbreviations: OR, odds ratios; CI, confidence interval; BMI, body mass index; BPWAI, photoplethysmography waveform area index; Ref: reference.
